# Molecular phylogeny of Triatomini (Hemiptera: Reduviidae: Triatominae)

**DOI:** 10.1186/1756-3305-7-149

**Published:** 2014-03-31

**Authors:** Silvia Andrade Justi, Claudia A M Russo, Jacenir Reis dos Santos Mallet, Marcos Takashi Obara, Cleber Galvão

**Affiliations:** 1 Departamento de Genética, Laboratório de Biologia Evolutiva Teórica e Aplicada, Universidade Federal do Rio de Janeiro, CCS, Instituto de Biologia, Rio de Janeiro, Brazil; 2 Laboratório de Transmissores de Leishmanioses, Instituto Oswaldo Cruz, Fundação Oswaldo Cruz, Rio de Janeiro, Brazil; 3 Faculdade de Ceilândia, Universidade de Brasília, Brasília, Brazil; 4 Laboratório Nacional e Internacional de Referência em Taxonomia de Triatomíneos, Instituto Oswaldo Cruz, Fundação Oswaldo Cruz, Rio de Janeiro, Brazil

**Keywords:** Triatomini, Species complex, Monophyly

## Abstract

**Background:**

The Triatomini and Rhodniini (Hemiptera: Reduviidae) tribes include the most diverse Chagas disease vectors; however, the phylogenetic relationships within the tribes remain obscure. This study provides the most comprehensive phylogeny of Triatomini reported to date.

**Methods:**

The relationships between all of the Triatomini genera and representatives of the three Rhodniini species groups were examined in a novel molecular phylogenetic analysis based on the following six molecular markers: the mitochondrial 16S; Cytochrome Oxidase I and II (COI and COII) and Cytochrome B (Cyt B); and the nuclear 18S and 28S.

**Results:**

Our results show that the *Rhodnius prolixus* and *R. pictipes* groups are more closely related to each other than to the *R. pallescens* group. For Triatomini, we demonstrate that the large complexes within the paraphyletic *Triatoma* genus are closely associated with their geographical distribution. Additionally, we observe that the divergence within the *spinolai* and *flavida* complex clades are higher than in the other *Triatoma* complexes.

**Conclusions:**

We propose that the *spinolai* and *flavida* complexes should be ranked under the genera *Mepraia* and *Nesotriatoma*. Finally, we conclude that a thorough morphological investigation of the paraphyletic genera *Triatoma* and *Panstrongylus* is required to accurately assign queries to natural genera.

## Background

Chagas disease, or American Trypanosomiasis, is one of the 10 most seriously neglected tropical diseases [1]. It currently affects nine million people [2], and more than 70 million people live under a serious risk of infection [3]. This vector-borne disease is transmitted by triatomine bugs (kissing bugs) infected with the parasite *Trypanosoma cruzi*[4]. All 148 described species of the Triatominae subfamily (Hemiptera: Reduviidae) are considered potential Chagas disease vectors [5,6].

The Triatominae subfamily includes 15 genera, seven of which comprise the Triatomini tribe, the most diverse, and two of which are assigned to the Rhodniini tribe, the second most diverse concerning species number [6]. In the most recent taxonomic review of this group, the authors suggested synonymisation of the genera *Meccus*, *Mepraia* and *Nesotriatoma* with *Triatoma*, which is the most diverse genus of the subfamily. The generic status of these groups has been under contention because there is no consensus on whether each group constitutes a species complex or a genus [5-9].

The genus *Triatoma* is diverse in terms of the number of species (it includes 82) [6,10,11] and morphology. This diversity has led to the division of *Triatoma* into complexes based on their morphological similarities and geographic distributions [6-9], but no formal cladistic analysis has been performed to corroborate the assignment of these groups.

Although species complexes are not formally recognized as taxonomic ranks and, thus, do not necessarily represent natural groups, we propose that they should be monophyletic. This statement is tightly linked to the idea that once the relationships between vector species are known, information about a species may be reliably extrapolated to other closely related species [12]. Previous molecular phylogenetic studies have shown that some *Triatoma* complexes are not monophyletic [13,14]. However, most of these molecular analyses were based on a single specimen per species and a single molecular marker.

The Rhodniini tribe comprises two genera: *Rhodnius* (18 species) and *Psammolestes* (three species), the former being divided into three species groups, namely, *pallescens, prolixus* and *pictipes*[15]. Although the relationship between these groups has not yet been established, with results in the literature conflicting [13,16], it seems that *Rhodnius* is a paraphyletic lineage, with *Psammolestes* being closely related to the *prolixus* group [16].

In this study, we investigated which groups (genera and species complexes) within Triatomini constitute natural groups. To this end, we conducted a comprehensive molecular phylogenetic analysis of Triatomini, pioneering the inclusion of all Triatomini genera, many specimens per species and several markers per sample. We also included representatives of the three Rhodniini groups to further test ingroup monophyly. The results enabled us to accurately classify the higher groups within the Triatomini tribe, to identify monophyletic genera and complexes and to pinpoint which of these groups should be subjected to a rigorous morphological review to accurately assign natural groups.

## Methods

### Taxon sampling

The sampling strategy applied in this study aimed to include specimens from different populations representing the largest possible diversity of Triatomini to test the validity of current taxonomic assignments. A total of 104 specimens representing 54 Triatomini species were included, including sequences available in GenBank. To further test ingroup monophyly, we also included 10 Rhodniini species. *Stenopoda* sp. (Stenopodainae: Reduviidae), a member of a distinct subfamily of Reduviidae [17], was selected as the outgroup. The employed Triatominae nomenclature followed the most recently published review on the subfamily [6].

Voucher specimens for all of the adult samples sequenced in this study were deposited in the Herman Lent Triatominae Collection (CT-IOC) at the *Instituto Oswaldo Cruz, FIOCRUZ*. All the information about the specimens can be found in Table 1. Some of the obtained specimens consisted of first-instar nymphs, eggs or adult legs. These specimens were not deposited in the collection because the entire sample was used for DNA extraction. Nevertheless, the identification of these specimens was reliable because they were obtained from laboratory colonies with known identities of the parental generation.

**Table 1:** Specimens examined, including laboratory colony source, locality information (when available), voucher depository, ID (unique specimen identifier number), and GenBank accession numbers

**Species**	**ID**	**Voucher number**	**Source**	**Geographic origen**	**Marker**					
					**COI**	**COII**	**CytB**	**16S**	**28S**	**18S**
*D. maxima*	92	3465	LDP	México	KC249306	-	KC249226	KC248968	KC249134	KC249092
	186	3520	LaTec	El Triunfo, México	KC249305	KC249399	KC249225	KC248967	-	-
*E.mucronatus*	-	-	GenBank	-	-	-	-	JQ897794	JQ897635	JQ897555
*H. matsunoi*	106	-	LNIRTT		-	KC249400	-	-	-	-
*Linshcosteus sp.*	-	-	GenBank	-	-	-	-	AF394595	-	-
*P. geniculatus*	-	-	GenBank	-	-	-	-	AF394593	-	-
*P. lignarius*	-	-	GenBank	-	AF449141	-	-	AY185833	-	-
*P. lutzi*	202	3524	LTL	Santa Quitéria, CE, Brazil	KC249307	KC249401	KC249227	KC248969	KC249135	-
*P. megistus*	128	3463	LACEN	Nova Prata, RS, Brazil	KC249308	KC249402	KC249228	KC248970	KC249136	-
	129	3476	LACEN	Boa Vista do Cadeado, RS, Brazil	KC249309	-	KC249229	KC248971	KC249137	-
	130	3477	LACEN	Tres Passos, RS, Brazil	-	-	KC249230	KC248972	KC249138	-
	131	3478	LACEN	Salvador do Sul, RS, Brazil	KC249310	-	KC249231	KC248973	KC249139	-
	132	3479	LACEN	Barão do Triunfo, RS, Brazil	KC249311	-	-	KC248974	KC249140	-
	183	3517	LaTec	Pitangui, MG, Brazil	KC249312	KC249403	KC249232	KC248975	KC249141	-
*P. tupynambai*	127	3462	LACEN	Dom Feliciano, RS, Brazil	-	-	KC249233	KC248977	-	-
	138	3485	LACEN	Pinheiro Machado, RS, Brazil	-	KC249404	KC249234	KC248978	KC249142	-
*Paratriatoma hirsuta*	-	-	GenBank	-	-	-	-	FJ230443	-	-
*R. brethesi*	197	3426	LNIRTT	Acará River, AM, Brazil	KC249313	KC249405	KC249235	KC248980	-	-
*R. colombiensis*	-	-	GenBank	-	-	-	FJ229360	AY035438	-	-
*R. domesticus*	-	-	GenBank	-	-	-	-	AY035440	-	-
*R. ecuadoriensis*	-	-	GenBank	-	-	GQ869665	-	-	-	-
*R. nasutus*	-	-	GenBank	-	-	-	-	-	AF435856	-
*R. neivai*	-	-	GenBank	-	AF449137	-	-	-	-	-
*R. pallescens*	-	-	GenBank	-	-	-	EF071584	-	-	-
*R. pictipes*	200	3429	LNIRTT	Bega, Abaetetuba, PA, Brazil	KC249315	KC249408	-	KC248982	-	KC249094
*R. prolixus*	-	-	GenBank	-	AF449138	-	-	-	AF435862	AY345868
*R. stali*	195	3424	LNIRTT	Alto Beni, Bolivia	KC249316	KC249409	KC249236	KC248983	-	-
*Stenopoda sp*	-	-	GenBank	-	-	-	-	FJ230414	FJ230574	FJ230493
*T. brasiliensis*	40	3384	LNIRTT	Curaçá, BA, Brazil	KC249319,KC249320	KC249415,KC249416	KC249240	KC248986	-	-
	41	3385	LNIRTT	Sobral, CE, Brazil	-	-	KC249241	KC248987	-	-
	174	3510	LaTec	Tauá, CE, Brazil	KC249318	KC249413	KC249239	KC248985	KC249145	-
*T. breyeri*	56	-	IIBISMED	Mataral, Cochabamba, Bolivia	KC249321	KC249417	KC249242	KC248988	-	-
*T. bruneri*	98	3468	LNIRTT	Cuba	-	KC249418	-	KC248989	KC249146	-
*T. carcavalloi*	78	3395	LNIRTT	São Gerônimo, RS, Brazil	KC249322	KC249419	KC249244	KC248991	-	KC249097
*T. circummaculata*	120	-	LNIRTT	Caçapava do Sul, RS, Brazil	KC249323	KC249421	-	KC248992	KC249147	KC249098
	121	-	LACEN	Piratini, RS, Brazil	KC249324	KC249422	-	KC248993	-	-
	122	3473	LACEN	Piratini, RS, Brazil	KC249325	-	KC249245	KC248994	KC249148	KC249099
	126	3461	LACEN	Dom Feliciano, RS, Brazil	-	-	-	KC248996	-	-
*T. costalimai*	35	3381	LNIRTT	Posse, GO, Brazil	KC249327,KC249328	KC249425	KC249246	KC248997	-	KC249101
	42	-	IIBISMED	Chiquitania, Cochabamba, Bolivia	KC249329	KC249426	KC249247	KC248998	KC249149	-
*T. delpontei*	53	-	IIBISMED	Chaco Tita, Cochabamba, Bolivia	KC249330	KC249427	KC249248	KC248999	-	-
*T. dimidiata*	20	3444	LaTec	**-**	KC249335	KC249431	-	KC249004	KC249152	-
	94	3466	LNIRTT	Central América	KC249336,KC249337	KC249432	-	KC249005	KC249155	-
	100	3470	LNIRTT	México	KC249333	-	-	KC249002	-	-
*T. eratyrusiformis*	-	-	GenBank	-	GQ336898	-	JN102360	AY035466	-	-
*T. flavida*	-	-	GenBank	-	-	-	-	AY035451	-	AJ421959
*T. garciabesi*	89	3405	LNIRTT	Rivadaria, Argentina	KC249338	-	KC249249	KC249006	KC249158	KC249102
*T. guasayana*	55	-	IIBISMED	Chaco Tita, Cochabamba, Bolivia	KC249342	-	KC249251	KC249010	-	-
	82	3398	LNIRTT	Santa Cruz, Bolívia	KC249343	KC249438	KC249252	KC249011	KC249162	KC249103
*T. guazu*	29	3455	LNIRTT	Barra do Garça, MT, Brazil	-	KC249440	-	KC249013	KC249164	KC249105
*T. infestans*	58	-	IIBISMED	Cotapachi, Cochabamba, Bolivia	KC249349	KC249442	KC249256	KC249015	KC249168	KC249109
	60	-	IIBISMED	Mataral, Cochabamba, Bolivia	KC249351	KC249443	KC249257	KC249016	KC249169	KC249107
	62	-	IIBISMED	Ilicuni, Cochabamba, Bolivia	KC249353	KC249445	KC249259	KC249018	-	-
	63	-	IIBISMED	Ilicuni, Cochabamba, Bolivia	KC249354	KC249446	KC249260	KC249019	-	-
	66	3386	LNIRTT	Guarani das Missões, RS, Brazil	-	-	-	KC249021	-	-
	68	3388	LNIRTT	Argentina	-	-	-	KC249023	-	-
	69	3389	LNIRTT	Montevideo, Uruguai	-	KC249447	KC249262	KC249024	KC249172	-
	44	-	IIBISMED	Chaco Tita Cochabamba	KC249346	-	KC249255	KC249025	KC249166	KC249108
*T. juazeirensis*	209	3430	LTL	Uiabí, BA, Brazil	-	-	KC249263	KC249026	KC249173	-
*T. jurbergi*	30	3456	LNIRTT	Alto Garça MT, Brazil	-	KC249448	KC249264	KC249027	KC249174	KC249110
*T. klugi*	75	3393	LNIRTT	Nova Petrópolis, RS, Brazil	KC249356	KC249449	KC249265	KC249028	-	-
*T. lecticularia*	151	3411	LaTec	-	-	KC249450	-	KC249029	KC249175	KC249111
*T. longipennis*	26	3450	LaTec	-	-	KC249453	KC249267	KC249032	-	-
	97	3467	LNIRTT	México	KC249358	-	-	KC249033	-	-
	165	3501	LaTec	México	KC249357	KC249452	-	KC249031	KC249177	-
*T. maculata*	203	3525	LTL	Água Fria, RR, Brazil	-	KC249454	-	KC249034		-
*T. matogrossensis*	31	3374	LNIRTT	Bahia, Brazil	KC249361	KC249458	-	KC249038	-	-
	32	3375	LNIRTT	Aquidauana , MS, Brazil	-	KC249459	KC249271	KC249039	KC249181	-
	33	3377	LNIRTT	Alegria, MT, Brazil	-	KC249460	KC249272	KC249040	KC249182	KC249114
	192	3423	LTL	São Gabriel D’oeste, MS, Brazil	KC249360	KC249457	KC249270	KC249037	KC249180	KC249113
*T. mazzottii*	-	-	GenBank	-	DQ198805	-	DQ198816	AY035446	-	AJ243333
*T. melanica*	-	3447	LaTec	-	-	KC249461	-	KC249041	KC249183	-
*T. melanosoma*	70	3390	LNIRTT	Missiones Argentina	KC249362	-	KC249273	KC249042	-	-
*T. nitida*	-	-	GenBank	-	-	-	AF045723	AF045702	-	-
*T. pallidipennis*	18	3442	LaTec	-	-	-	-	KC249045	-	-
*T. phyllosoma*	-	-	GenBank	-	DQ198806	-	DQ198818	-	-	AJ243329
*T. picturata*	-	-	GenBank	-	-	-	DQ198817	AY185840	-	AJ243332
*T. platensis*	96	-	LNIRTT	Montevideo Uruguai	-	-	KC249274	KC249047	KC249186	-
*T. protracta*	93	3407	LNIRTT	Monte Diablo, California, EUA	-	KC249463	-	KC249048	KC249187	-
*T. pseudomaculata*	34	3379	LNIRTT	Curaçá, BA, Brazil	-	-	-	KC249057	KC249196	-
	211	3432	LTL	Várzea Alegre, CE, Brazil	KC249364	KC249464	KC249275	KC249050	KC249189	-
	212	3433	LTL	Várzea Alegre, CE, Brazil	-	KC249465	KC249276	KC249051	KC249190	-
	214	3435	LTL	Várzea Alegre, CE, Brazil	KC249365	KC249467	KC249277	KC249053	KC249192	-
*T. recurva*	-	-	GenBank	-	DQ198803	-	DQ198813	FJ230417	-	FJ230496
*T. rubrofasciata*	-	-	GenBank	-	-	-	-	AY127046	-	AJ421960
*T. rubrovaria*	76	3459	LNIRTT	Caçapava do Sul, RS, Brazil	KC249375	KC249477	KC249286	KC249066	-	-
	77	3394	LNIRTT	Quevedos, RS, Brazil	KC249376	-	KC249287	KC249067	KC249204	KC249122
	156	3416	LaTec	Canguçu, RS, Brazil	KC249374	KC249476	KC249285	KC249065	KC249203	KC249121
	123	3474	LACEN	Piratini, RS, Brazil	KC249369	KC249470	-	KC249058	KC249197	KC249116
	134	3481	LACEN	Canguçu, RS, Brazil	KC249370	KC249471	KC249281	KC249059	KC249198	KC249117
	136	3483	LACEN	Pinheiro Machado, RS, Brazil	KC249372	KC249473	KC249283	KC249061	KC249200	KC249119
	140	3487	LACEN	Canguçu, RS, Brazil	KC249373	KC249475	-	KC249064	KC249202	KC249120
*T. sanguisuga*	-	-	GenBank	-	-	JF500886	HQ141317|	AF045696	-	-
*T. sherlocki*	80	3396	LNIRTT	-	KC249377	KC249478	KC249288	KC249068	KC249205	-
*T. sordida*	38	3382	LNIRTT	Rondonópolis, MT, Brazil	-	KC249479	-	KC249071	-	-
	46	-	IIBISMED	Romerillo, Cochabamba, Bolivia	KC249379,KC249380	KC249480	-	KC249072	KC249207	-
	47	-	IIBISMED	Romerillo, Cochabamba, Bolivia	KC249381,KC249382	-	KC249290	KC249073	KC249208	KC249124
	83	3399	LNIRTT	La Paz, Bolívia	KC249383	KC249481	KC249291	KC249074	KC249209	-
	85	3401	LNIRTT	Pantanal, MS, Brazil	KC249384	KC249482	KC249292	KC249075	KC249210	KC249125
	86	3402	LNIRTT	Santa Cruz, Bolívia	KC249385	-	KC249293	KC249076	KC249211	-
	88	3404	LNIRTT	San Miguel Corrientes, Argentina	KC249387	KC249484	KC249295	KC249078	KC249213	-
	90	3406	LNIRTT	Poconé, MT, Brazil	KC249388	-	-	KC249079	-	-
*Triatoma sp.*	50	-	IIBISMED	Mataral, Cochabamba, Bolivia	KC249339	KC249435	-	KC249007	KC249159	-
*T. spinolai*	-	-	GenBank	-	GQ336893	-	JN102358	AF324518	-	AJ421961
*T. tibiamaculata*	79	3460	LNIRTT	-	KC249390	KC249486	KC249297	KC249081	KC249215	-
	177	3513	LaTec	Mogiguaçu, RS, Brazil	KC249389	KC249485	KC249296	KC249080	KC249214	KC249127
*T. vandae*	28	3452	LNIRTT	Pantanal, MT, Brazil	KC249391	KC249487	KC249298	KC249082	KC249216	KC249128
	73	3392	LNIRTT	Rio Verde do Mato Grosso, MT, Brazil	KC249392	KC249488	KC249299	KC249083	KC249217	KC249129
	74	3458	LNIRTT	Rondonópolis, MT, Brazil	KC249393,KC249394	KC249489	KC249300	KC249084	KC249218	-
*T. vitticeps*	81	3397	LNIRTT	-	KC249396	KC249491	KC249303	KC249087	KC249220	KC249132
	91	-	LTL	Rio de Janeiro, Brazil	KC249397	KC249492	KC249304	KC249088	KC249221	-
	168	3504	LaTec	Itanhomi, MG, Brazil	KC249395	KC249490	KC249301	KC249085	-	KC249130
*T. williami*	36	-	LNIRTT	-	-	KC249493	-	KC249089	-	-
*T. wygodzynski*	17	3441	LaTec	-	KC249398	KC249494	-	KC249090	KC249222	KC249133
	205	3527	LTL	Sta Rita de Caldas, MG, Brazil	-	-	-	KC249091	-	-

LTL - Laboratório de Transmissores de Leishmanioses, IOC, FIOCRUZ; LaTec - Laboratório de Triatomíneos e epidemiologia da Doença de Chagas, CPqRR, FIOCRUZ; LACEN - Laboratório Central, Rio Grande do Sul, Ministério da Saúde; IIBISMED - Instituto de Investigaciones Biomédicas, Facultad de Medicina, Universidad Mayor de San Simón, Cochabamba, Bolivia.

### DNA extraction, amplification and sequencing

The DNA extraction was performed using the protocol described by Aljanabi and Martinez [18] or using the Qiagen Blood and Tissue kit, according to the manufacturer’s recommendations. The following PCR cycling conditions were employed: 95°C for 5 min; 35 cycles of 95°C for 1 min, 49–45°C for 1 min, and 72°C for 1 min; and 72°C for 10 min. The sequences of the primers used for amplification are shown in Table 2. The reaction mixtures contained 10 mM Tris–HCl/50 mM KCl buffer, 0.25 mM dNTPs, 10 μM forward primer, 10 μM reverse primer, 3 mM MgCl_2_, 2.5 U of Taq polymerase and 10–30 ng of DNA. The primers used to amplify the mitochondrial COI, COII, CytB and 16S and the nuclear ribosomal 18S and 28S markers are listed in Table 2.

**Table 2:** Primers used in this study

**Marker**	**Forward primer**	**Reverse primer**
COI	5′-GGTCAACAAATCATAAAGATATTGG-3′ [19]	5′-AAACTTCAGGGTGACCAAAAAATCA-3′ [19]
	5′-CCTGCAGGAGGAGGAGAYCC-3′ [20]	5′ - TAAGCGTCTGGGTAGTCTGARTAKCG-3′; [21]
	5′-ATTGRATTTTDAGTCATAGGGAG-3′ (this study)	5′-TATTYGTWTGATCDGTWGG-3′ (this study)
CytB	5′-GGACG(AT)GG(AT)ATTTATTATGGATC-3′ [22]	5′-ATTACTCCTCCTAGYTTATTAGGAATT-3′ [23]
COII	5′-ATGATTTTAAGCTTCATTTATAAAGAT-3′ [23]	5′-GTCTGAATATCATATCTTCAATATCA-3′ [23]
16S	5′-CGCCTGTTTATCAAAAACAT-3′ [24]	5′-CTCCGGTTTGAACTCAGATCA-3′ [24]
28S	5′- AGTCGKGTTGCTTGAKAGTGCAG-3′ [25]	5′- TTCAATTTCATTKCGCCTT-3′ [25]
	5′-CTTTTAAATGATTTGAGATGGCCTC-3′ (this study)	-
18S	5′-AAATTACCCACTCCCGGCA-3′ [24]	5′-TGGTGUGGTTTCCCGTGT T-3′ [24]

The PCR-amplified products were purified using the ExoSAP-IT (USB® products), according to the manufacturer’s recommendations, and both strands were subsequently sequenced. The sequencing reactions were performed using the ABI Prism® BigDye® Terminator v3.1 Cycle Sequencing kit (Applied Biosystems), with the same primers employed for PCR, in ABI 3130 and ABI 3730 sequencers (PDTIS Platform, FIOCRUZ and the Genetics Department of UFRJ, respectively). The obtained sequences were assembled using MEGA 4.0 [26] and SeqMan Lasergene v. 7.0 (DNAStar, Inc.) software.

### Sequence alignments and molecular datasets

Different approaches were used to align the coding sequences and the ribosomal DNA markers. The coding sequences were translated and then aligned using ClustalW [27] implemented in MEGA 4.0 [26] software. The ribosomal DNA sequences were aligned using MAFFT [28] with the Q-INS-I option, which takes the secondary RNA structure into consideration.

We first constructed an alignment including all the sequences obtained (Additional file 1: Table S1), but there was too much missing data in this matrix, which included 169 individuals. To minimise the effect of missing data on the analysis, a new alignment was constructed based on the above method with the aim of maximising diversity, considering that each taxon in the dataset had to be comparable to all others, that is, all specimens must include comparable sequences.

The final individual alignments were concatenated by name using SeaView [29], generating a matrix including 115 individuals and 6,029 nucleotides (Table 1). This dataset is available on the Dryad database (http://datadryad.org/) and upon request.

### Phylogenetic analyses

jModeltest [30] was used to assess the best fit model for each of the markers. The markers CytB, COII, 18S and 28S fit models less parametric than GTR + Γ (data not shown). Despite this fact, GTR + Γ was used for all the markers as this is the next best model available in the programs used. The use of a more parametric model is supported by the fact that the application of a model less parametric than the “real” model leads to a strong accentuation of errors in the recovered tree [31].

The Maximum Likelihood (ML) tree was obtained through a search of 200 independent runs with independent parsimony starting trees using RAxML 7.0.4 [32]. The alignment was partitioned by marker, and for each partition, the gamma parameter was estimated individually, coupled to the GTR model. To assess the reliability of the recovered clades, 1,000 bootstrap [33] replicates were performed using the rapid bootstrap algorithm implemented in RaxML.

Additionally, a Bayesian approach was applied to reconstruct the phylogeny of the concatenated dataset using MrBayes 3 [34]. The data were also partitioned based on markers, and GTR + Γ (four categories) was used separately for each partition, with the gamma parameter being estimated individually. The trees were sampled every 1,000 generations for 100 million generations in two independent runs with four chains each. *Burn-in* was set to 50% of the sampled trees.

## Results

The recovered phylogenies (ML and BI) yielded very similar trees, with the generated clades supporting their agreement with one another.

### The Rhodniini tribe

**Figure 1:**
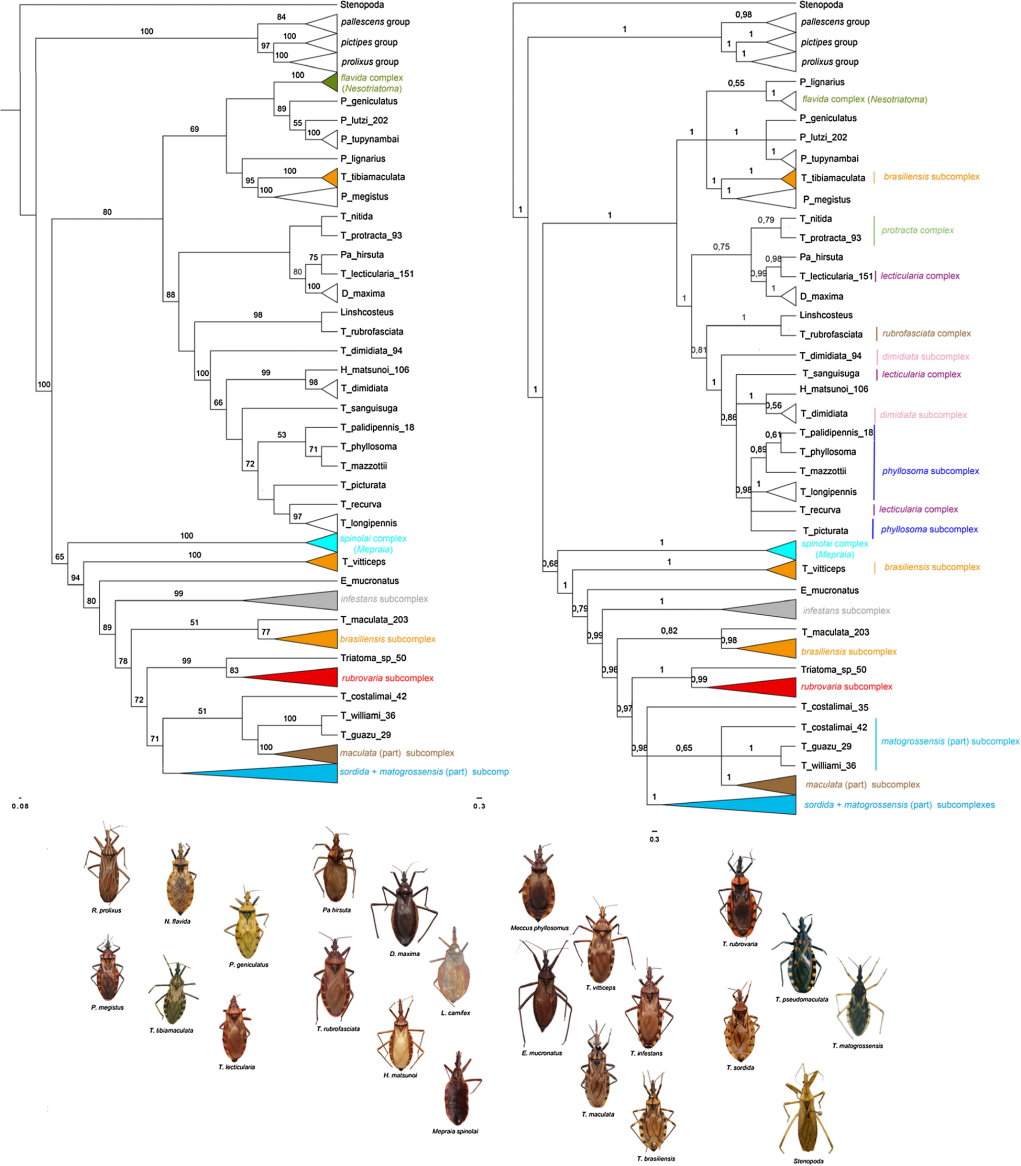
**Best ML tree (on the left) and Bayesian consensus tree (on the right) reconstructed.** Bars on the right highlight the non-monophyletic groups. Numbers above branches represent clade support higher than 50 and 0.5, respectively. Triatominae photos by Carolina Dale. *Stenopoda spinulosa* photo by Brad Barnd. Photos are not to scale.

The Rhodniini tribe (Figure 1, Additional file 2: Figure S1 and Additional file 3: Figure S2) was recovered with high support (BS = 100, PP = 1), as were most relationships within the tribe. The *prolixus* group was recovered as a sister taxon to the *pictipes* group (BS = 97, PP = 1), and these groups form a sister clade to the *pallescens* group. The only species that could not be confidently placed within its clade was *R. neivai*, which was recovered within the *prolixus* group as a sister species to *R. nasutus*, but support was lower (BS = 80, PP = 0.7; Figure 1, Additional file 2: Figure S1 and Additional file 3: Figure S2).

### The Triatomini tribe

The Triatomini tribe was recovered with the highest support (BS = 100, PP = 1). The tribe was shown to be divided into three main lineages: Clade (1), *Panstrongylus* + the *flavida* complex (*Nesotriatoma*) + *T. tibiamaculata* (BS = 69, PP = 1); Clade (2), the monotypic genera (*Hermanlentia*, *Paratriatoma*, *Dipetalogaster*) + *Linshcosteus* + Northern Hemisphere *Triatoma* (BS = 88, PP = 1); and Clade (3), Southern Hemisphere *Triatoma* (including the *spinolai* complex or *Mepraia*) and *Eratyrus* (BS = 65, PP = 0.68).

#### Clade (1): Panstrongylus + the flavida complex (Nesotriatoma) + T. tibiamaculata

The *flavida* complex (*Nesotriatoma*) was recovered with the highest support in all three phylogenies, showing a close relationship with the clade formed by *P. geniculatus* + *P. lutzi* + *P. tupynambai. P. megistus* was placed as a sister taxon to *T. tibiamaculata* (BS = 95, PP = 1); while *P. lignarius* could not be confidently placed in the clade (BS < 50, PP = 0.55).

#### Clade (2): the monotypic genera (Hermanlentia, Paratriatoma, Dipetalogaster) + Linshcosteus + Northern Hemisphere Triatoma

In this clade, the phylogenies showed close relationships among *Paratriatoma (Pa.)*, *Dipetalogaster* and *T. nitida*, *T. protracta* (*protracta* complex) and *T. lecticularia* (*lecticularia* complex). *Pa. hirsuta* was always recovered as a sister species to *T. lecticularia* (BS = 75, PP = 0.98), and this pair was sister to *D. maxima* (BS = 80, PP = 0.99). The indicated species from the *protracta* complex were always recovered as a single clade that was closely related to *D. maxima*, *Pa. hirsuta* and *T. lecticularia*.

The tropicopolitan *T. rubrofasciata* species was recovered as a sister species to *Linshcosteus* in both phylogenies with high support (BS = 98, PP = 1). This pair of species is closely related to the clade formed by the *dimidiata* subcomplex + *T. sanguisuga* (*lecticularia* subcomplex) + *Hermanlentia matsunoi* + the *phyllosoma* subcomplex + *T. recurva* (BS = 100, PP = 1).

*H. matsunoi* appeared as a sister taxon to *T. dimidiata* from Mexico with high support (BS = 99, PP = 1). The *phyllosoma* subcomplex was not recovered as monophyletic as *T. recurva* was recovered close to *T. longipennis*, although the bootstrap for this clade was not high (BS = 72, PP = 0.98).

#### Clade (3) Southern Hemisphere Triatoma and Eratyrus

This clade was formed by the *spinolai* complex and the species assigned to the *infestans* complex, which were not recovered as monophyletic. The *spinolai* complex was recovered as monophyletic (BS = 100, PP = 1) in both phylogenies, and as sister taxa to the *infestans* complex.

*T. vitticeps* was recovered as a sister taxon to *E. mucronatus* and to the remaining Southern Hemisphere *Triatoma* subcomplexes of the *infestans* (BS = 94, PP = 1)*.* The *infestans* and *rubrovaria* subcomplexes were recovered as monophyletic (BS = 99, PP = 1 and BS = 83, PP = 0.99, respectively). In addition, the *rubrovaria* subcomplex was closely related to a short-winged *Triatoma* sp. (BS = 99, PP = 1) that resembles *T. guasayana*, which was discovered in the bromeliads of the Bolivian Chaco by F. Noireau.

*T. maculata* was not closely related to the other species of the *maculata* subcomplex. This taxon clustered with the *brasiliensis* subcomplex (BS = 51, PP = 0.82), except for *T. tibiamaculata* and *T. vitticeps*, which clustered elsewhere. The remaining species of the *maculata* subcomplex clustered in a large clade with the *sordida* and *matogrossensis* subcomplexes (BS = 71, PP = 0.98).

## Discussion

### Phylogenetic analyses

The reconstructed phylogenies presented in this report showed similar topologies and consistent branch support values. The posterior probability values were almost always higher than the bootstrap values, as expected [31] (Additional file 2: Figure S1 and Additional file 3: Figure S2).

Nonetheless, deep relationships, such as those between complexes, could be resolved. In addition, relationships within the *infestans* subcomplexes remain unclear (Additional file 2: Figure S1 and Additional file 3: Figure S2). The short terminal branches of these subcomplexes indicate that their diversification must have occurred recently. Under this scenario, incomplete lineage sorting would account for the lack of phylogenetic resolution within the group [35].

A different approach will be adopted in future studies to assess the relationships between closely related species that could not be resolved here. New unlinked nuclear markers, especially those linked to development and reproduction [36], will be sequenced to generate a species tree reconstruction [37], which is a more suitable method of phylogenetic reconstruction for closely related species.

### The Rhodniini tribe

The Rhodniini tribe comprises only 2 genera: *Rhodnius* and *Psammolestes. Rhodnius* has long been known to be easily distinguishable from other Triatominae, but the morphological discrimination of the species within *Rhodnius* is rather difficult [38]. Moreover, there is no uncertainty in the literature regarding the species groups assigned within *Rhodnius*; the uncertainty is related to the relationships between these groups.

Previously described molecular phylogenies of these genera have yielded distinct results. For instance, Lyman *et al.*[16] showed the *pallescens* group to be more closely related to the *pictipes* group, but Hypsa *et al.*[13] found the *pictipes* group to be closer to the *prolixus* group, which is consistent with our results. This difference could be due to differences in taxon sampling rather than differences in the gene trees, as both of these authors used mitochondrial markers. In this work, the taxon sampling process included a larger number of species than were included by Lyman *et al.*[16] (see also [13]). Wiens and Tiu [39] demonstrated that the addition of taxa should improve the accuracy of a phylogenetic reconstruction. The amount of data (less than 10% of the size of our alignment) from Hypsa *et al.*[13] was overturned by their taxon sampling, which included twice the number of species as the first work.

### The Triatomini tribe

The Triatomini tribe is the most diverse tribe within the subfamily, and many taxonomic proposals have been put forth for the groups belonging to this tribe. The most prominent of these proposals is that *Meccus*, *Mepraia* and *Nesotriatoma* be considered as genera or species complexes belonging to *Triatoma*[5,6,8]. Dujardin *et al.*[40] noted these confusing systematics with another example: the number of monotypic genera within the tribe and the number of subspecies (at times also considered separate species) assigned to Triatomini. Figure 1, based on our results, highlights the most accepted Triatomini groups that are not monophyletic. We show that *Triatoma* and *Panstrongylus* are not natural groups. However, diversities formerly placed under the generic names *Mepraia* and *Nesotriatoma*, but not *Meccus,* consist of monophyletic lineages*.*

Therefore, based on our results, we indicate that *Mepraia* and *Nesotriatoma* should be ranked as genera, as previously proposed [5]. The branch lengths of the reconstructed phylogenies (Figure 1, Additional file 2: Figure S1 and Additional file 3: Figure S2) showed much greater distances between the species assigned to each of these genera than within the other *Triatoma* complexes. In addition, if the species belonging to *Nesotriatoma* are considered a species complex of another genus, it is reasonable to include these species in the genus *Panstrongylus*.

Previous studies have indicated a putative paraphyletic status for *Panstrongylus*, despite a lack of resolution in some groups [13,14,41,42]. In our topology, *Panstrongylus* is clearly divided into two groups: one including *P. tupynambai*, *P. lutzi* and *P. geniculatus* as sister taxa to *Nesotriatoma* and another group showing a close and highly supported relationship between *T. tibiamaculata* and *P. megistus*.

The most prominent morphological characteristic that separates *Panstrongylus* from other Triatomini is the short head of these species, with antennae close to the eyes [8]. The non-monophyletic status of *Panstrongylus* (Figure 1; see also [14]) indicates that this putative diagnostic characteristic of the genus might be a morphological convergence. Indeed, some *Panstrongylus* populations show variation in eye size according to their habitat, and this variation influences the distances between the antennae and the eyes [43]. *Panstrongylus* species tend to present *Triatoma*-like head shapes [43] during development when the nymphs exhibit smaller eyes. Furthermore, North American *Triatoma* may display smaller heads and antennae that are closer to the eyes than their South American counterpart*s*[6].

*Triatoma* is composed of two distinct paraphyletic groups: one occurring in the Northern hemisphere and the other in the Southern Hemisphere; one exception found in the present work was *T. tibiamaculata*, which clusters with *Panstrongylus* elsewhere. The previous assignments of *Triatoma* species into complexes took into consideration the geographical distributions of the groups and their morphological features (e.g. [6]). Our results clearly indicate that monophyletic clades of *Triatoma* species, which do not necessarily correspond to these complexes, are correlated with restricted geographical distributions corresponding to different biogeographical provinces [44]. This is particularly evident in South America.

#### Northern Hemisphere Triatoma and the less diverse genera

*T. lecticularia* is sister to *Pa. hirsuta*. This pair of species is closely related to *D. maxima*, which is a genus whose head shape resembles a large *Triatoma*. Furthermore, *Pa. hirsuta* exhibits a head shape similar to *T. lecticularia*, which was observed by Lent and Wygodzinsky [8].

*H. matsunoi*, which was included in a phylogenetic study for the first time in the present work, was recovered as the sister taxon to the Mexican lineage of *T. dimidiata. H. matsunoi* was first described as belonging to *Triatoma*[45] based on the main features used to characterise the Triatomini genera. Subsequently, Jurberg and Galvão [46] found major differences in the male genitalia of this species relative to other Triatomini and reassigned it to a new monotypic genus.

*T. rubrofasciata* appears to be the species that is closest to *Linshcosteus*, which is the only Triatomini genus exclusively from the Old World, more precisely, from India. Although we did not include Old World *Triatoma* in our analyses, previous morphometric analyses have shown *Linshcosteus* to be distinct from Old world *Triatoma* and from the closely related species *T. rubrofasciata* from the New World [47].

The *dimidiata* subcomplex was not recovered as a natural group because the two sampled *T. dimidiata* s.l. lineages [48] did not cluster, and the clade also included *T. lecticularia* and *H. matsunoi*. Consistent with our results, Espinoza *et al.*[49] recently published a reconstructed phylogeny showing the relationships among the North American *Triatoma* species. They included *T. gerstaeckeri* and *T. brailovskyi* (not included here) in their analysis and demonstrated the close relationships between these species and those from the *dimidiata* and *phyllosoma* subcomplexes, confirming the need to review these groups.

#### Southern Hemisphere Triatoma

Most subcomplexes assigned to the *infestans* complex were not recovered as monophyletic. The only natural groups recovered were the *infestans* and *rubrovaria* subcomplexes.

As noted above, most of the monophyletic clades recovered for these *Triatoma* can be associated with a South American biogeographical province. This shows that geographical distribution currently has greater importance than morphology in the process of assigning natural groups to the genus. Henceforth, the geographical provinces (related to biomes) will be referred to as described in Morrone [44].

*T. vitticeps*, the first *Triatoma* lineage to diverge in this clade, is found in the Atlantic Forest and shares morphological similarities with the unsampled species *T. melanocephala*[6], which is a rare species found exclusively in northeastern Brazil [50]. Although both species were assigned to the former *brasiliensis* complex ([6]), both our results and the number of sex chromosomes in these species, which differs from the other Southern Hemisphere *Triatoma*, would exclude them from this group [50].

The next lineage to diverge in this clade was *Eratyrus mucronatus*. The genus *Eratyrus* differs from *Triatoma* in displaying a long spine-shaped posterior process of the scutellum and a long first rostral segment, which is nearly as long as the second segment [8]. Although we did not include *E. cuspidatus* in our analysis, the morphology of this genus is rather distinct, and apart from its phylogenetic position within *Triatoma*, this species is not a subject of “systematic dispute” in the literature.

*Triatoma maculata* appears as the sister taxon to part of the *brasiliensis* subcomplex (except *T. tibiamaculata* and *T. vitticeps*). Previous studies have demonstrated the close relationships among some species in the *brasiliensis* subcomplex [51]. However, these studies did not include *T. maculata* in their analyses. In contrast, an earlier study revealed a possible close relationship between *T. brasiliensis* and *T. maculata*[13]. *T. maculata* is exclusively found in the Amazonian forest, while the *brasiliensis* subcomplex is exclusive to the Caatinga province in northeastern Brazil.

The species assigned to the *infestans*, *sordida*, and *rubrovaria* subcomplexes currently exhibit overlapping distributions as they all occur in the Chacoan dominion. The *infestans* subcomplex was found to be monophyletic, with its distribution occurring mainly in Chaco province. It is important to highlight that only sylvatic populations were considered for this designation because *T. infestans* shows a distribution related to human migration in most Southern American countries [52].

The *Triatoma* sp. informally described by François Noireau as a short-winged form of *T. guasayana* appears as the sister taxon to the *rubrovaria* subcomplex. This previously undescribed species was collected in Chaco province from bromeliads, which form a different microhabitat than the rock piles in which *rubrovaria* species are usually found [53]. Conversely, the *rubrovaria* subcomplex is restricted to Pampa province and the Paraná dominion. As Pampa and Chaco provinces belong to the Chacoan dominion, *Triatoma* sp. and the *rubrovaria* complex inhabit historically related areas [44], we predict that microhabitat adaptations account for the morphological divergence observed between these groups.

The most morphologically diverse clade includes species from the *sordida*, *maculata* (except for *T. maculata*) and *matogrossensis* subcomplexes. This is also the most widespread group in South America and occupies most of Cerrado and Chaco provinces.

## Conclusions

Our results show that a thorough evolutionary mapping of the morphological characteristics of Triatomini is long overdue. For example, head shape, which was previously used to distinguish *Panstrongylus* from *Triatoma*, does not appear to be a reliable characteristic; the highly supported *P. megistus* + *T. tibiamaculata* sister taxa corroborate this conclusion.

In addition, the only published cladistic analysis of a Triatominae group, for *Panstrongylus*[8], does not agree with our results, though this might be due to the fact that *Nesotriatoma* and *T. tibiamaculata* were not included in their analysis. We have shown that the genus *Triatoma* and a majority of the *Triatoma* species complexes are not monophyletic. Knowledge of morphologies and the evolutionary histories of morphological traits are imperative in assigning natural groups. In the case of Triatomini, such knowledge is particularly relevant due to the epidemiological importance of these organisms [12].

## Competing interests

The authors declare that they have no competing interests.

## Authors’ contributions

SAJ designed the study, acquired data (specimen acquisition and sequencing), performed all the analyses, interpreted the results, and drafted and reviewed the manuscript. CAMR designed the study, acquired data (specimen acquisition), interpreted the results and reviewed the manuscript. JRSM acquired data (specimen acquisition), interpreted the results and reviewed the manuscript. MTO acquired data (specimen acquisition), interpreted the results and reviewed the manuscript. CG designed the study, acquired data (specimen acquisition), interpreted the results and reviewed the manuscript. All authors read and approved the final version of the manuscript.

## Supplementary Material

Additional file 1: Table S1 All specimens obtained, including laboratory colony source, locality information (when available), voucher depository, ID (unique specimen identifier number),and GenBank accessionnumbers. LTL - Laboratório de Transmissores de Leishmanioses, IOC, FIOCRUZ; LaTec - Laboratório de Triatomíneos e epidemiologia da Doença de Chagas, CPqRR, FIOCRUZ; LACEN - Laboratório Central, Rio Grande do Sul, Ministério da Saúde; IIBISMED - Instituto de Investigaciones Biomédicas, Facultad de Medicina, Universidad Mayor de San Simón, Cochabamba, Bolivia. Click here for file

Additional file 2: Figure S1 The best ML tree obtained. The numbers above branches refer to bootstrap values. Click here for file

Additional file 3: Figure S2 The Bayesian consensus tree obtained. The burn-in was set at 50% of the sampled trees, and the posterior probabilities are shown above branches. Click here for file
