# Supplementary material for: Molecular phylogeny of Triatomini (Hemiptera: Reduviidae: Triatominae)
Source: Parasit Vectors. 2014 Mar 31;7:149. doi: 10.1186/1756-3305-7-149 (PMC4021723; doi:10.1186/1756-3305-7-149)
Supplement: Additional file 1: Table S1 — All specimens obtained, including laboratory colony source, locality information (when available), voucher depository, ID (unique specimen identifier number),and GenBank accessionnumbers. LTL - Laboratório de Transmissores de Leishmanioses, IOC, FIOCRUZ; LaTec - Laboratório de Triatomíneos e epidemiologia da Doença de Chagas, CPqRR, FIOCRUZ; LACEN - Laboratório Central, Rio Grande do Sul, Ministério da Saúde; IIBISMED - Instituto de Investigaciones Biomédicas, Facultad de Medicina, Universidad Mayor de San Simón, Cochabamba, Bolivia. [file 1756-3305-7-149-S1.doc]

**Additional file 1: Table S1: All specimens obtained, including laboratory colony source, locality information (when available), voucher depository, ID (unique specimen identifier number),and GenBank accessionnumbers. LTL - Laboratório de Transmissores de Leishmanioses, IOC, FIOCRUZ; LaTec - Laboratório de Triatomíneos e epidemiologia da Doença de Chagas, CPqRR, FIOCRUZ; LACEN - Laboratório Central, Rio Grande do Sul, Ministério da Saúde; IIBISMED - Instituto de Investigaciones Biomédicas, Facultad de Medicina, Universidad Mayor de San Simón, Cochabamba, Bolivia.**

| **Species** | **ID** | **Voucher Number** | **Source** | **Geographic Origen** | **Marker** | | | | | |
| --- | --- | --- | --- | --- | --- | --- | --- | --- | --- | --- |
|  |  |  |  |  | **COI** | **COII** | **CytB** | **16S** | **28S** | **18S** |
| *D. máxima* | 92 | 3465 | LDP | México | KC249306 | - | KC249226 | KC248968 | KC249134 | KC249092 |
|  | 186 | 3520 | LaTec | El Triunfo, México | KC249305 | KC249399 | KC249225 | KC248967 | - | - |
| *E.mucronatus* | - | - | GenBank | - | - | - | - | JQ897794 | JQ897635 | JQ897555 |
| *H. matsunoi* | 106 | - | LNIRTT |  | - | KC249400 | - | - | - | - |
| *Linshcosteus sp.* | - | - | GenBank | - | - | - | - | AF394595 | - | - |
| *P. geniculatus* | - | - | GenBank | - | - | - | - | AF394593 | - | - |
| *P. lignarius* | - | - | GenBank | - | AF449141 | - | - | AY185833 | - | - |
| *P. lutzi* | - | - | GenBank | - | - | - | - | AY035449 | - | - |
|  | 202 | 3524 | LTL | Santa Quitéria, CE, Brazil | KC249307 | KC249401 | KC249227 | KC248969 | KC249135 | - |
| *P. megistus* | 128 | 3463 | LACEN | Nova Prata, RS, Brazil | KC249308 | KC249402 | KC249228 | KC248970 | KC249136 | - |
|  | 129 | 3476 | LACEN | Boa Vista do Cadeado, RS, Brazil | KC249309 | - | KC249229 | KC248971 | KC249137 | - |
|  | 130 | 3477 | LACEN | Tres Passos, RS, Brazil | - | - | KC249230 | KC248972 | KC249138 | - |
|  | 131 | 3478 | LACEN | Salvador do Sul, RS, Brazil | KC249310 | - | KC249231 | KC248973 | KC249139 | - |
|  | 132 | 3479 | LACEN | Barão do Triunfo, RS, Brazil | KC249311 | - | - | KC248974 | KC249140 | - |
|  | 201 | 3523 | LTL | Tanguá, RJ, Brazil | - | - | - | KC248976 | - | - |
|  | 183 | 3517 | LaTec | Pitangui, MG, Brazil | KC249312 | KC249403 | KC249232 | KC248975 | KC249141 | - |
| *P. tupynambai* | 127 | 3462 | LACEN | Dom Feliciano, RS, Brazil | - | - | KC249233 | KC248977 | - | - |
|  | 138 | 3485 | LACEN | Pinheiro Machado, RS, Brazil | - | KC249404 | KC249234 | KC248978 | KC249142 | - |
| *P. rufotuberculatus* | - | - | GenBank | - | - | - | - | - | - | AJ421955 |
| *Paratriatoma hirsuta* | - | - | GenBank | - | - | - | - | FJ230443 | - | - |
| *R. brethesi* | 197 | 3426 | LNIRTT | Acará River, AM, Brazil | KC249313 | KC249405 | KC249235 | KC248980 | - | - |
|  | 198 | 3427 | LNIRTT | Acará River, AM, Brazil | - | KC249406 | - | KC248979 | - | - |
| *R. colombiensis* | - | - | GenBank | - | - | - | FJ229360 | AY035438 | - | - |
| *R. domesticus* | - | - | GenBank | - | - | - | - | AY035440 | - | - |
| *R. ecuadoriensis* | - | - | GenBank | - | - | GQ869665 | - | - | - | - |
| *R. nasutus* | - | - | GenBank | - | - | - | - | - | AF435856 | - |
| *R. neivai* | - | - | GenBank | - | AF449137 | - | - | - | - | - |
| *R. pallescens* | - | - | GenBank | - | - | - | EF071584 | - | - | - |
| *R. pictipes* | 199 | 3428 | LNIRTT | Bega, Abaetetuba, PA, Brazil | KC249314 | KC249407 | - | KC248981 | - | KC249093 |
|  | 200 | 3429 | LNIRTT | Bega, Abaetetuba, PA, Brazil | KC249315 | KC249408 | - | KC248982 | - | KC249094 |
| *R. prolixus* | - | - | GenBank | - | AF449138 | - | - | - | AF435862 | AY345868 |
| *R. stali* | 195 | 3424 | LNIRTT | Alto Beni, Bolivia | KC249316 | KC249409 | KC249236 | KC248983 | - | - |
|  | 196 | 3425 | LNIRTT | Alto Beni, Bolivia | KC249317 | KC249410 | KC249237 | KC248984 | - | KC249095 |
| *R.neglectus* | - | - | GenBank | - | - | - | AF045716 | - | - | - |
| *Stenopoda sp* | - | - | GenBank | - | - | - | - | FJ230414 | FJ230574 | FJ230493 |
| *T. baratai* | 189 | - | LNIRTT | Corumbá, MS, , Brazil | - | KC249411 | KC249238 | - | KC249143 | - |
|  | 190 | - | LNIRTT | Corumbá, MS, , Brazil | - | KC249412 | - | - | KC249144 | - |
| *T. barberi* | - | - | GenBank | - | - | - | - | - | - | AJ421958 |
| *T. bassolsae* | - | - | GenBank | - | - | - | AY859410 | - | - | - |
| *T. brasiliensis* | 39 | 3383 | LNIRTT | Mossoró, RN, Brazil | - | KC249414 | - | - | - | - |
|  | 40 | 3384 | LNIRTT | Curaçá, BA, Brazil | KC249319,KC249320 | KC249415,KC249416 | KC249240 | KC248986 | - | - |
|  | 41 | 3385 | LNIRTT | Sobral, CE, Brazil | - | - | KC249241 | KC248987 | - | - |
|  | 174 | 3510 | LaTec | Tauá, CE, Brazil | KC249318 | KC249413 | KC249239 | KC248985 | KC249145 | - |
| *T. breyeri* | 56 | - | IIBISMED | Mataral, Cochabamba, Bolivia | KC249321 | KC249417 | KC249242 | KC248988 | - | - |
| *T. bruneri* | 98 | 3468 | LNIRTT | Cuba | - | KC249418 | - | KC248989 | KC249146 | - |
| *T. carcavalloi* | 78 | 3395 | LNIRTT | São Gerônimo, RS, Brazil | KC249322 | KC249419 | KC249244 | KC248991 | - | KC249097 |
|  | 141 | 3410 | LACEN | São Jerônimo, RS, Brazil | - | - | KC249243 | KC248990 | - | KC249096 |
| *T. circummaculata* | 120 | - | LNIRTT | Caçapava do Sul, RS, Brazil | KC249323 | KC249421 | - | KC248992 | KC249147 | KC249098 |
|  | 121 | - | LACEN | Piratini, RS, Brazil | KC249324 | KC249422 | - | KC248993 | - | - |
|  | 122 | 3473 | LACEN | Piratini, RS, Brazil | KC249325 | - | KC249245 | KC248994 | KC249148 | KC249099 |
|  | 124 | 3475 | LACEN | Canguçu, RS, Brazil | - | KC249423 | - | - | - | - |
|  | 125 | - | LACEN | Alegrete, RS, Brazil | KC249326 | KC249424 | - | KC248995 | - | KC249100 |
|  | 126 | 3461 | LACEN | Dom Feliciano, RS, Brazil | - | - | - | KC248996 | - | - |
| *T. costalimai* | 35 | 3381 | LNIRTT | Posse, GO, Brazil | KC249327,KC249328 | KC249425 | KC249246 | KC248997 | - | KC249101 |
|  | 42 | - | IIBISMED | Chiquitania, Cochabamba, Bolivia | KC249329 | KC249426 | KC249247 | KC248998 | KC249149 | - |
| *T. delpontei* | 53 | - | IIBISMED | Chaco Tita, Cochabamba, Bolivia | KC249330 | KC249427 | KC249248 | KC248999 | - | - |
|  | 71 | 3457 | LNIRTT | Santa Cruz, Bolívia | KC249331 | KC249428 | - | KC249000 | KC249150 | - |
|  | 72 | 3391 | LNIRTT | Santiago Delstero, Argentina | KC249332 | KC249429 | - | KC249001 | KC249151 | - |
| *T. dimidiata* | 20 | 3444 | LaTec | **-** | KC249335 | KC249431 | - | KC249004 | KC249152 | - |
|  | 21 | 3445 | LaTec | **-** | - | - | - | - | KC249153 | - |
|  | 22 | 3446 | LaTec | México | - | - | - | - | KC249154 | - |
|  | 94 | 3466 | LNIRTT | Central América | KC249336,KC249337 | KC249432 | - | KC249005 | KC249155 | - |
|  | 100 | 3470 | LNIRTT | México | KC249333 | - | - | KC249002 | - | - |
|  | 171 | 3507 | LaTec | México | KC249334 | KC249430 | - | KC249003 | - | - |
| *T. eratyrusiformis* | - | - | GenBank | - | GQ336898 | - | JN102360 | AY035466 | - | - |
| *T. flavida* | - | - | GenBank | - | - | - | - | AY035451 | - | AJ421959 |
| *T. gajardoi* | - | - | GenBank | - | GQ336895 | - | JN102359 | - | - | - |
| *T. garciabesi* | 6 | 3439 | LNIRTT | Rivadaria,Argentina | - | KC249433 | - | - | KC249156 | - |
|  | 7 | 3440 | LNIRTT | Rivadaria, Argentina | - | KC249434 | - | - | KC249157 | - |
|  | 89 | 3405 | LNIRTT | Rivadaria, Argentina | KC249338 | - | KC249249 | KC249006 | KC249158 | KC249102 |
| *T. guasayana* | 54 | - | IIBISMED | Chaco Tita, Cochabamba, Bolivia | KC249341 | KC249437 | KC249250 | KC249009 | KC249161 | - |
|  | 55 | - | IIBISMED | Chaco Tita, Cochabamba, Bolivia | KC249342 | - | KC249251 | KC249010 | - | - |
|  | 82 | 3398 | LNIRTT | Santa Cruz, Bolívia | KC249343 | KC249438 | KC249252 | KC249011 | KC249162 | KC249103 |
|  | 84 | 3400 | LNIRTT | Chaco Tita, Bolivia | KC249344,KC249345 | KC249439 | KC249253 | KC249012 | KC249163 | KC249104 |
| *T. guazu* | 29 | 3455 | LNIRTT | Barra do Garça, MT, Brazil | - | KC249440 | - | KC249013 | KC249164 | KC249105 |
| *T. infestans* | 58 | - | IIBISMED | Cotapachi, Cochabamba, Bolivia | KC249349 | KC249442 | KC249256 | KC249015 | KC249168 | KC249109 |
|  | 59 | - | IIBISMED | - | KC249350 | - | - | - | - | - |
|  | 60 | - | IIBISMED | Mataral, Cochabamba, Bolivia | KC249351 | KC249443 | KC249257 | KC249016 | KC249169 | KC249107 |
|  | 61 | - | IIBISMED | Mataral, Cochabamba, Bolivia | KC249352 | KC249444 | KC249258 | KC249017 | KC249170 | - |
|  | 62 | - | IIBISMED | Ilicuni, Cochabamba, Bolivia | KC249353 | KC249445 | KC249259 | KC249018 | - | - |
|  | 63 | - | IIBISMED | Ilicuni, Cochabamba, Bolivia | KC249354 | KC249446 | KC249260 | KC249019 | - | - |
|  | 64 | - | IIBISMED | Callejas, Cochabamba, Bolivia | KC249355 | - | KC249261 | KC249020 | KC249171 | - |
|  | 66 | 3386 | LNIRTT | Guarani das Missões, RS, Brazil | - | - | - | KC249021 | - | - |
|  | 67 | 3387 | LNIRTT | Entre Juis, RS, Brazil | - | - | - | KC249022 | - | - |
|  | 68 | 3388 | LNIRTT | Argentina | - | - | - | KC249023 | - | - |
|  | 69 | 3389 | LNIRTT | Montevideo, Uruguai | - | KC249447 | KC249262 | KC249024 | KC249172 | - |
|  | 162 | 3498 | LaTec | Vila Brasília, GO, Brazil | KC249348 | KC249441 | KC249254 | KC249014 | KC249165 | KC249106 |
|  | 44 | - | IIBISMED | Chaco Tita Cochabamba | KC249346 | - | KC249255 | KC249025 | KC249166 | KC249108 |
|  | 45 | - | IIBISMED | Chaco Tita Cochabamba | KC249347 | - | - | - | KC249167 | - |
| *T. juazeirensis* | 209 | 3430 | LTL | Uiabí, BA, Brazil | - | - | KC249263 | KC249026 | KC249173 | - |
| *T. jurbergi* | 30 | 3456 | LNIRTT | Alto Garça MT, Brazil | - | KC249448 | KC249264 | KC249027 | KC249174 | KC249110 |
| *T. klugi* | 75 | 3393 | LNIRTT | Nova Petrópolis, RS, Brazil | KC249356 | KC249449 | KC249265 | KC249028 | - | - |
| *T. lecticularia* | 95 | 3408 | LNIRTT | Walrika, Oklahoma, EUA | - | KC249451 | KC249266 | KC249030 | KC249176 | - |
|  | 151 | 3411 | LaTec | - | - | KC249450 | - | KC249029 | KC249175 | KC249111 |
| *T. longipennis* | 26 | 3450 | LaTec | - | - | KC249453 | KC249267 | KC249032 | - | - |
|  | 97 | 3467 | LNIRTT | México | KC249358 | - | - | KC249033 | - | - |
|  | 165 | 3501 | LaTec | México | KC249357 | KC249452 | - | KC249031 | KC249177 | - |
| *T. maculata* | 203 | 3525 | LTL | Água Fria, RR, Brazil | - | KC249454 | - | KC249034 |  | - |
|  | 210 | 3431 | LTL | Agua Fria, RR, Brazil | - | KC249455 | KC249268 | KC249035 | KC249178 | - |
| *T. matogrossensis* | 31 | 3374 | LNIRTT | Bahia, Brazil | KC249361 | KC249458 | - | KC249038 | - | - |
|  | 32 | 3375 | LNIRTT | Aquidauana , MS, Brazil | - | KC249459 | KC249271 | KC249039 | KC249181 | - |
|  | 33 | 3377 | LNIRTT | Alegria, MT, Brazil | - | KC249460 | KC249272 | KC249040 | KC249182 | KC249114 |
|  | 191 | 3422 | LTL | São Gabriel D'oeste, MS, Brazil | KC249359 | KC249456 | KC249269 | KC249036 | KC249179 | KC249112 |
|  | 192 | 3423 | LTL | São Gabriel D'oeste, MS, Brazil | KC249360 | KC249457 | KC249270 | KC249037 | KC249180 | KC249113 |
| *T. mazzottii* | - | - | GenBank | - | DQ198805 | - | DQ198816 | AY035446 | - | AJ243333 |
| *T. melanica* | - | 3447 | LaTec | - | - | KC249461 | - | KC249041 | KC249183 | - |
| *T. melanosoma* | 70 | 3390 | LNIRTT | Missiones Argentina | KC249362 | - | KC249273 | KC249042 | - | - |
| *T. mexicana* | - | - | GenBank | - | DQ198807 | - | DQ118976 | - | - | - |
| *T. nítida* | - | - | GenBank | - | - | - | AF045723 | AF045702 | - | - |
| *T. pallidipennis* | 18 | 3442 | LaTec | - | - | - | - | KC249045 | - | - |
|  | 101 | 3471 | LNIRTT | Mexico | - | - | - | KC249043 | - | - |
|  | 159 | 3516 | LaTec | Oaxtepec, México | - | - | - | KC249044 | KC249184 | KC249115 |
| *T. phyllosoma* | - | - | GenBank | - | DQ198806 | - | DQ198818 | - | - | AJ243329 |
| *T. picturata* | - | - | GenBank | - | - | - | DQ198817 | AY185840 | - | AJ243332 |
| *T. platensis* | 96 | - | LNIRTT | Montevideo Uruguai | - | - | KC249274 | KC249047 | KC249186 | - |
|  | 154 | - | LaTec | Argentina | KC249363 | KC249462 | - | KC249046 | KC249185 | - |
| *T. protracta* | 93 | 3407 | LNIRTT | Monte Diablo, California, EUA | - | KC249463 | - | KC249048 | KC249187 | - |
| *T. pseudomaculata* | 34 | 3379 | LNIRTT | Curaçá, BA, Brazil | - | - | - | KC249057 | KC249196 | - |
|  | 207 | - | LTL | Bom Jesus, PB, Brazil | - | - | - | KC249049 | KC249188 | - |
|  | 211 | 3432 | LTL | Várzea Alegre, CE, Brazil | KC249364 | KC249464 | KC249275 | KC249050 | KC249189 | - |
|  | 212 | 3433 | LTL | Várzea Alegre, CE, Brazil | - | KC249465 | KC249276 | KC249051 | KC249190 | - |
|  | 213 | 3434 | LTL | Várzea Alegre, CE, Brazil | - | KC249466 | - | KC249052 | KC249191 | - |
|  | 214 | 3435 | LTL | Várzea Alegre, CE, Brazil | KC249365 | KC249467 | KC249277 | KC249053 | KC249192 | - |
|  | 215 | 3436 | LTL | Várzea Alegre, CE, Brazil | KC249366 | KC249468 | KC249278 | KC249054 | KC249193 | - |
|  | 216 | 3437 | LTL | Várzea Alegre, CE, Brazil | KC249367 | - | KC249279 | KC249055 | KC249194 | - |
|  | 217 | 3438 | LTL | Várzea Alegre, CE, Brazil | KC249368 | KC249469 | KC249280 | KC249056 | KC249195 | - |
| *T. recurva* | - | - | GenBank | - | DQ198803 | - | DQ198813 | FJ230417 | - | FJ230496 |
| *T. rubida cochimiensis* | - | - | GenBank | - | DQ198802 | - | DQ198811 | - | - | - |
| *T. rubida sonoriana* | - | - | GenBank | - | DQ198800 | - | DQ198810 | - | - | - |
| *T. rubida uhleri* | - | - | GenBank | - | DQ198801 | - | DQ198809 | - | - | - |
| *T. rubrofasciata* | - | - | GenBank | - | - | - | - | AY127046 | - | AJ421960 |
| *T. rubrovaria* | 76 | 3459 | LNIRTT | Caçapava do Sul, RS, Brazil | KC249375 | KC249477 | KC249286 | KC249066 | - | - |
|  | 77 | 3394 | LNIRTT | Quevedos, RS, Brazil | KC249376 | - | KC249287 | KC249067 | KC249204 | KC249122 |
|  | 156 | 3416 | LaTec | Canguçu, RS, Brazil | KC249374 | KC249476 | KC249285 | KC249065 | KC249203 | KC249121 |
|  | 123 | 3474 | LACEN | Piratini, RS, Brazil | KC249369 | KC249470 | - | KC249058 | KC249197 | KC249116 |
|  | 134 | 3481 | LACEN | Canguçu, RS, Brazil | KC249370 | KC249471 | KC249281 | KC249059 | KC249198 | KC249117 |
|  | 135 | 3482 | LACEN | Canguçu, RS, Brazil | KC249371 | KC249472 | KC249282 | KC249060 | KC249199 | KC249118 |
|  | 136 | 3483 | LACEN | Pinheiro Machado, RS, Brazil | KC249372 | KC249473 | KC249283 | KC249061 | KC249200 | KC249119 |
|  | 137 | 3484 | LACEN | Pinheiro Machado, RS, Brazil | - | - | KC249284 | KC249062 | KC249201 | - |
|  | 139 | 3486 | LACEN | Canguçu, RS, Brazil | - | KC249474 | - | KC249063 | - | - |
|  | 140 | 3487 | LACEN | Canguçu, RS, Brazil | KC249373 | KC249475 | - | KC249064 | KC249202 | KC249120 |
| *T. sanguisuga* | - | - | GenBank | - | - | JF500886 | HQ141317| | AF045696 | - | - |
| *T. sherlocki* | 80 | 3396 | LNIRTT | - | KC249377 | KC249478 | KC249288 | KC249068 | KC249205 | - |
| *T. sórdida* | 38 | 3382 | LNIRTT | Rondonópolis, MT, Brazil | - | KC249479 | - | KC249071 | - | - |
|  | 46 | - | IIBISMED | Romerillo, Cochabamba, Bolivia | KC249379,KC249380 | KC249480 | - | KC249072 | KC249207 | - |
|  | 47 | - | IIBISMED | Romerillo, Cochabamba, Bolivia | KC249381,KC249382 | - | KC249290 | KC249073 | KC249208 | KC249124 |
|  | 83 | 3399 | LNIRTT | La Paz, Bolívia | KC249383 | KC249481 | KC249291 | KC249074 | KC249209 | - |
|  | 85 | 3401 | LNIRTT | Pantanal, MS, Brazil | KC249384 | KC249482 | KC249292 | KC249075 | KC249210 | KC249125 |
|  | 86 | 3402 | LNIRTT | Santa Cruz, Bolívia | KC249385 | - | KC249293 | KC249076 | KC249211 | - |
|  | 87 | 3403 | LNIRTT | Posse, GO, Brazil | KC249386 | KC249483 | KC249294 | KC249077 | KC249212 | KC249126 |
|  | 88 | 3404 | LNIRTT | San Miguel Corrientes, Argentina | KC249387 | KC249484 | KC249295 | KC249078 | KC249213 | - |
|  | 90 | 3406 | LNIRTT | Poconé, MT, Brazil | KC249388 | - | - | KC249079 | - | - |
|  | 180 | 3419 | LaTec | Ibiracatu, MG, Brazil | KC249378 | - | KC249289 | KC249069 | KC249206 | KC249123 |
|  | 204 | 3526 | LTL | Parnaíba, MS, Brazil | - | - | - | KC249070 | - | - |
| *Triatoma sp.* | 50 | - | IIBISMED | Mataral, Cochabamba, Bolivia | KC249339 | KC249435 | - | KC249007 | KC249159 | - |
|  | 51 | - | IIBISMED | Mataral, Cochabamba, Bolivia | KC249340 | KC249436 | - | KC249008 | KC249160 | - |
| *T. spinolai* | - | - | GenBank | - | GQ336893 | - | JN102358 | AF324518 | - | AJ421961 |
| *T. tibiamaculata* | 79 | 3460 | LNIRTT | - | KC249390 | KC249486 | KC249297 | KC249081 | KC249215 | - |
|  | 177 | 3513 | LaTec | Mogiguaçu, RS, Brazil | KC249389 | KC249485 | KC249296 | KC249080 | KC249214 | KC249127 |
| *T. vandae* | 28 | 3452 | LNIRTT | Pantanal, MT, Brazil | KC249391 | KC249487 | KC249298 | KC249082 | KC249216 | KC249128 |
|  | 73 | 3392 | LNIRTT | Rio Verde do Mato Grosso, MT, Brazil | KC249392 | KC249488 | KC249299 | KC249083 | KC249217 | KC249129 |
|  | 74 | 3458 | LNIRTT | Rondonópolis, MT, Brazil | KC249393KC249394 | KC249489 | KC249300 | KC249084 | KC249218 | - |
| *T. vitticeps* | 19 | 3443 | LaTec | Itanhomi, MG, Brazil | - | - | KC249302 | KC249086 | KC249219 | KC249131 |
|  | 81 | 3397 | LNIRTT | - | KC249396 | KC249491 | KC249303 | KC249087 | KC249220 | KC249132 |
|  | 91 | - | LTL | Rio de Janeiro, Brazil | KC249397 | KC249492 | KC249304 | KC249088 | KC249221 | - |
|  | 168 | 3504 | LaTec | Itanhomi, MG, Brazil | KC249395 | KC249490 | KC249301 | KC249085 | - | KC249130 |
| *T. williami* | 36 | - | LNIRTT | - | - | KC249493 | - | KC249089 | - | - |
| *T. wygodzynski* | 17 | 3441 | LaTec | - | KC249398 | KC249494 | - | KC249090 | KC249222 | KC249133 |
|  | 205 | 3527 | LTL | Sta Rita de Caldas, MG, Brazil | - | - | - | KC249091 | - | - |
