# Supplementary figures and images for: Molecular phylogeny of Triatomini (Hemiptera: Reduviidae: Triatominae)
Source: Parasit Vectors. 2014 Mar 31;7:149. doi: 10.1186/1756-3305-7-149 (PMC4021723; doi:10.1186/1756-3305-7-149)

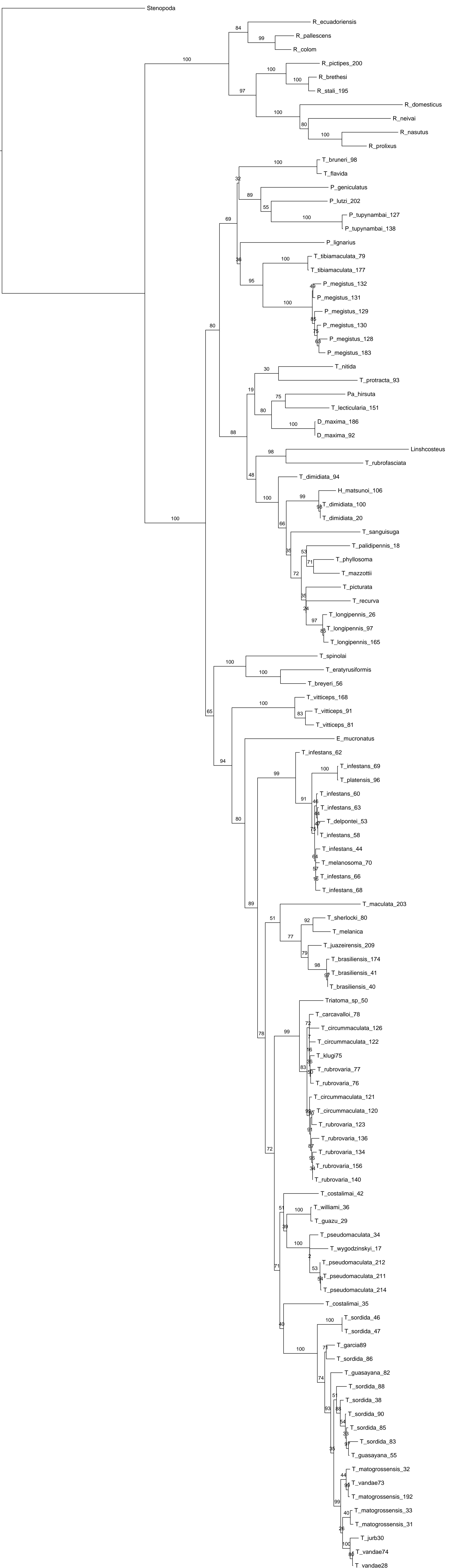

Supplement: Additional file 2: Figure S1 — The best ML tree obtained. The numbers above branches refer to bootstrap values. [file 1756-3305-7-149-S2.pdf]

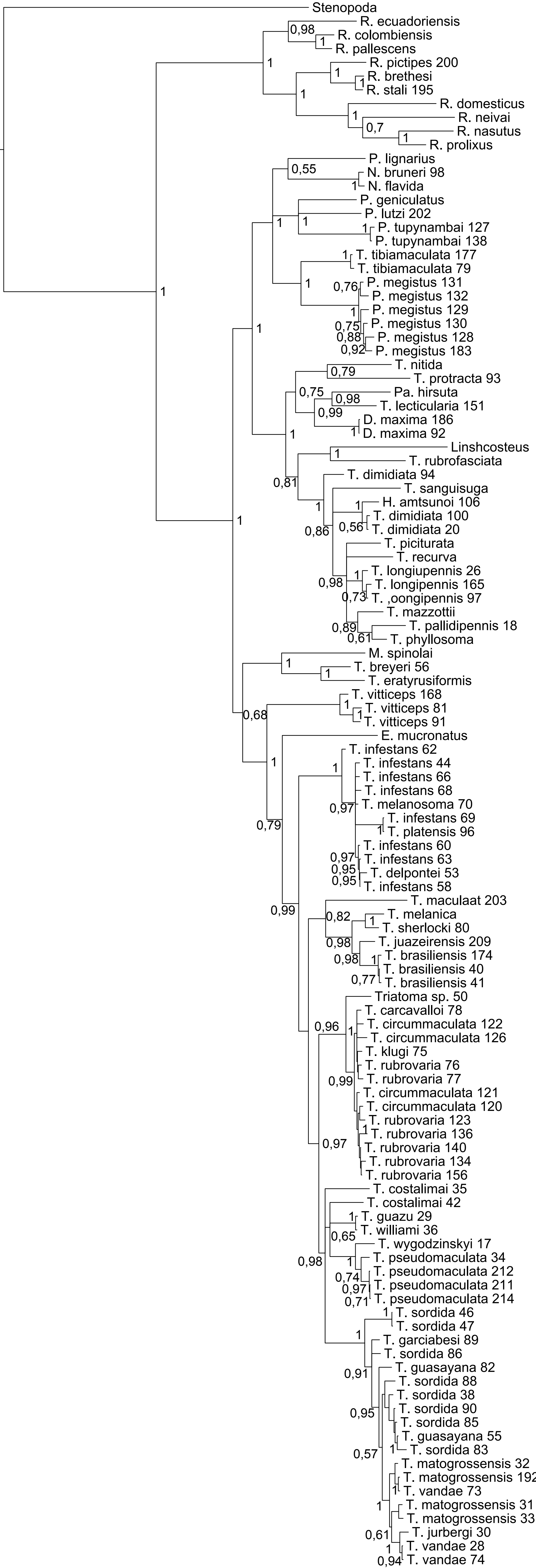

Supplement: Additional file 3: Figure S2 — The Bayesian consensus tree obtained. The burn-in was set at 50% of the sampled trees, and the posterior probabilities are shown above branches. [file 1756-3305-7-149-S3.pdf]
